# On the Nature and Treatment of Hydrocephalus Acutus

**Published:** 1819-01-01

**Authors:** 

**Affiliations:** Bristol


### *On the Nature and Treatment of Hydrocephalus Acutus.*

By Dr. PORTER, of Bristol.

> "*A man who works beyond the surface of things, though he may be wrong himself, yet he clears the way for others; and may chance to make even his errors subservient to the cause of truth*" BURKE.

MUCH has been written upon Medical Nomenclature, and many attempts have been made to improve it; but its first principle being erroneous, little advantage can be gained by its diversified or extended application.

So long as terms would arrogate to themselves the office of definitions, confusion must be the result. If terms in medicine and chemistry were absolute, like the symbols of algebraical quantity, we should not then be puzzled by discordant notions of agreement or disagreement between the name and the thing.

But as a system of nomenclature too simple will not suit the pride of science, we shall not reject the term Hydrocephalus; though it imply the effect only, and not the essence of the disease.

It is notorious, however, that this disease has been confounded with one of a chronic nature, which has nothing in common but the name, and that which the name implies; and on this account it were to be wished, that the term hydrocephalus had never been applied, or extended beyond the designation of the chronic hydrops cerebri; for to such a state of parts simply, it is alone appropriate; but as the adjunct *acutus* is sufficient to constitute the necessary distinction, and is now in general use, it has been adopted at the head of this paper.

In the investigation of this subject, we must set out with a pathological fact, which shows that one branch or more of any set of blood-vessels may be in that condition, which usually is expressed by the terms "inflammation of the part"—"great vascularity of the part"—"increased arterial action of the part," without the neighbouring vessels being altered from *their* healthy condition; and, that from the simple state of inflammatory condition of a single blood-vessel to that of vastly extended inflammation there exists every degree.

Now in hydrocephalus, as it is termed, I contend that the fatal link of the chain of morbid action consists of an inflammatory condition of the posterior arteries of the encephalon; I say the fatal link—the link on which the fate of the patient is suspended, and to which our attention should be directed; not the *mortal* link, by which the poor sufferer is dragged to the grave; and on which *Darwin* had his eye when he wrote—"Trephine!"

I have said that the inflammatory condition is confined to the posterior arteries of the encephalon; it is so. In hydrocephalus as such, the flushed face; the highly irritable iris; the frantic delirium, manifesting an inflammatory state of the facial branches of the external carotid and its meningeal arteries, are not symptoms necessarily present; they are only so when phrenitis is superadded; for it is an inflammatory state of the meningeal artery that produces the symptoms which range themselves under this term.

The symptoms attending the progress of hydrocephalus acutus, from its commencement to its termination, without the aid of morbid anatomy, would not direct us in the perception of its pure nature—"paulatim adoriens"—there is no violence until the mischief is irremediable; the inflammation, although seated at the fountain head of sensorial power, being sub-acute, proceeds, "lassitudine, febriculâ." He hangs his head, and reclines; he rubs the back of it on his mother's arm; he moans; he cannot bear to be touched; he knits his brows for a moment, at uncertain intervals; his face is pale, though his head is hot; the tongue is little furred, sometimes nearly clean; the stomach is sick, sometimes amounting to spontaneous vomiting; the bowels are torpid; and the pulse frequent. As the march of the disease advances, he puts his hand frequently to his head; one cheek flushes, the other remaining pale; he screams at intervals; he sometimes refers to his bowels, sometimes to his head, as the seat of pain; the pulse increases in frequency, but becomes unequal, beating a few strokes now and then slower than its mean ratio. The disease, for a few hours, or a day, assumes a deceitful aspect; the pulse falls; there is evidence of a change: I believe the effusion has begun, and soon finishing, the relief is of short duration. The pulse resumes its frequency; the pupils become dilated, one or both; strabismus ensues; stupor supervenes, not always unattended by convulsions; and death, after an uncertain number of hours, or days, from this period, closes the scene.

There is nothing in all this that would lead us to a knowledge of the true malady; these obvious symptoms are but so many effects of the inflammatory condition of the extended branches of the basilar artery, which we have been taught by dissection to recognize as the essential seat of the disease.

Much practical difficulty is found in diagnosis between the disease in question and infantile remittent fever. But, in the former, the tongue is much cleaner; in the latter, the child does not knit the brows. In the former, the child does not pick his nose; in the latter, there is not a sensible increase of heat in the head. Still infantile remittent fever may excite that condition of the vessels of the brain, which terminates in hydrocephalus; and such may be the effect of difficult dentition, or any constitutional or local irritation whatever.

This disease has also been confounded with phrenitis; and writers of reputation have chosen this term to express it, believing the diseases to be the same. To this there could not be much objection, had not the term a place in our nosologies from Sauvages to Macbride, inclusive; and these adopted as expressive of certain symptoms depending on an inflammatory condition of the branches of the external carotid arteries, exclusively; symptoms not necessarily present in hydrocephalus, but attending it, when an inflammatory condition of the meningeal is superadded to the morbid vascularity of the basilar artery. The disease being different *in situ,* and in symptoms, if not in nature, the terms should be different, if we admit the propriety of pleuritis, and the distinguishing term " pneumonitis;" for pleuritis is to the one, what pneumonitis is to the other.

If the highly celebrated Edinburgh Nosologist had been aware of the true nature of acute hydrocephalus, he would not have ventured to declare that phlegmasia of the brain cannot be distinguished from phlegmasia of its membranes. His own definitions of the two diseases in question, sufficiently distinguish them. "*Phrenitis;*" pyrexia vehemens; dolor capitis; rubor faciei; et occulorum lucis et soni intollerantia; pervigilium; delirium ferox vel typhomania." But as he did not apprehend hydrocephalus acutus to be a phlegmasia terminating in effusion, we find it in his nosology, a species in his genus "apoplexia." "Apoplexia hydrocephalica"—"paulatim adoriens; infantes et impubes primum lassitudine, febriculâ, et dolore capitis: dein pulsa tardiore, pupillæ dilatatione, et somnolentiâ, afficiens." If we admit these definitions to be correct, or nearly so, we cannot doubt or deny the distinct existence of phrenitis and hydrocephalus acutus. Yet, surprising as it should appear, the disease is not distinctly recognized any where in the last and best nosological work that has appeared in this or any other country; unless Mr. Good intends to comprehend it within his first species, "*Cephalitis,*" which ranges itself under the 7th genus of his order, *Hæmatica*. But if so, his definition goes too far; it will serve only to display the symptoms that phrenitis exhibits; symptoms, many of which are not displayed in hydrocephalus acutus. If the single species *cephalitis* were expunged, and in its place two species introduced, viz. *meningitis* and *encephalitis;* the former retaining for its definition that of *cephalitis,* the synonym of the more exceptionable term *phrenitis;* viz. "Acute pain in the head; intolerance of light and sound; face flushed; eyes red; cauma; watchfulness and delirium:"—the latter assuming for its definition, "chiefly affecting children; manifested by uneasiness of the head, drooping and reclining; slight aversion to light; brows knit at intervals; impatience of touch; pulse frequent, often unequal; bowels torpid; eyes not red; face not uniformly flushed; fever not ardent; somnolency; stupor;" we should then find hydrocephalus acutus in its proper place, and with a more significant nosological appellation, sufficiently distinguished from *phrenitis.*

In a note to empresma, Mr. Good has taken occasion to advert to the opinion of Dr. Cullen, as to the impracticability of distinguishing between inflammation of the brain and its membranes, and having the same view of the subject, he compresses all the phlegmasiæ of the encephalon within that of cephalitis, as Cullen had previously done under the term phrenitis. In the face of these two highly respectable authorities, I should feel dismayed in obtruding a contrary opinion on my medical brethren, were I not borne out by the general pathological fact, that inflammatory condition is not always extended to every set of vessels, or even to all the branches of the same vessel with which a part is furnished; and, that such is actually the fact in the diseases I would distinguish, not only the distinct symptoms they severally present during their continuance, but also the appearances they exhibit, after their termination in death, sufficiently demonstrate. Still it must be acknowledged, that they often glide imperceptibly into each other, as the inflammatory condition of the encephalon becomes more general. The extension of inflammation from one texture to another contiguous, obtains every where; but the confinement of inflammation to particular textures, and to particular vessels, previous to such extension, and very frequently without such extension, is equally true.

To avoid a false impression on the mind, with respect to the nature of inflammation, we should not employ the term "action," but "condition." If terms do not convey correct relative ideas, they are injurious to science; they are intended as a directing post to point out the way to the mind, by which it is to arrive at the idea of the thing spoken of. "Action" implies somewhat of increasing motion. Now, it has been demonstrated by physiologists, that motion is not always increased in inflammation, beyond the ordinary state of the vessels, but the reverse; and, therefore, is not essential to its existence. We sometimes see the vessels of a part extended as to capacity, and in consequence of that expansion, containing a much larger quantity of blood than vessels in their healthy state are wont to admit; and this state of expansion and fullness does exist prior to any increased action of the heart, and to any accelerated *vis a tergo* whatever. The single epithet "inflammation" is unobjectionable to express the condition of the diseased part; it states a fact, it does not offer an opinion. But observation has enabled us to discover, that the relative condition of the red vessels, with respect to that of their capillaries and exhalents, produces corresponding diversified phenomena; and that the condition of all these, with respect to the heart's action, produce other morbid phenomena, all of which range themselves very properly under the term "inflammation:" the former constitute local inflammation in all its varieties; the latter, every variety of cauma, or synocha. The varieties of local inflammation are, first, simple expansion and fullness of any set of vessels, including their capillaries, thereby accumulating blood, and extricating heat. This is the inflammation of gout, of rheumatism, of the exanthemata, and all inflammations liable to translation. It may have its source in irritation at some point remote from the obvious seat of the disease; by which, both the febrile action and peculiar inflammation are sympathetically induced: we should therefore be cautious in disturbing local inflammation of this character (the gutta rosacea inclusive) by topical applications. But the distending disposition of the artery and vein is not always extended equally to the capillaries, a resistance is set up at this point; the parts in consequence become turgid, and if unyeilding, a sense of violent pulsation is felt at each systole of the heart. The exhalents now suffer under an increased protrusion of sanguineous particles, more or less, according to their condition, and the relative condition of the continuous vessels, and the cardiac action incident to this state of things: this constitutes the most comprehensive variety of inflammation; and that to which alone many authors are disposed to attach the term. Under this state, the exhalents are pressed to effuse either the serum or the fibrin of the blood; or, to generate and effuse pus. On the yielding condition of the capillaries and exhalents, ceteris paribus, the termination of the disease depends: if the former expand, the disease terminates by resolution; if the latter only, by exudation, dependent for its nature on the structure of the part affected, and the violence of the heart's action. In this most extensive variety of inflammation, the source of irritation is in the part itself.

The intensity of the cauma is less or greater, according to the muscularity of the subject, state of plethora, and part labouring under inflammatory condition: and there is little or no disturbance of the cerebral or spinal functions, unless the brain or spine be the seat of disease; and this is vastly greatest in phrenitis. In typhoid fever, the cranial and spinal brains are primarily affected in their sensorial and locomotive function by the direct influence of contagion, and some other acknowledged remote causes, antecedent to vascular commotion: but in a subsequent stage of the disease, those organs not unfrequently suffer a further derangement, in common with many other parts of the body; having an increased liability, somewhat in the ratio of their more abundant vascularity: and here the essential cause of cauma rises up in typhus. And, if I am not mistaken, this last stage of obvious cerebral lesion, has been confounded with its less manifest primary derangement; and on these distinct conditions of cerebral disorder, contending pathologists have fixed their eye when disputing on the efficient cause of idiopathic fever.

Now the disease to which this Essay is directed, is a weak cauma, having its source in a sub-acute inflammatory condition of the base of the brain.

### *Cases and Dissections.*

Case I. April 7th, 1815. R. F. the child of a shoemaker, ætat: 17 months, has been drooping and moaning some weeks; head heavy, cannot hold it up; rubs the back of it frequently against the mother's arm; eyes look dull; it was sick yesterday; has refused food during the last week; bowels not constipated, having taken castor oil; will not suffer the pulse to be felt.

Calomel and jalap.

8th. Several scanty stools of mixed appearance; has not vomited yesterday or to-day; continues heavy; shews no aversion to light; cannot see its tongue; breath sweet; peevish; will not suffer himself to be touched.

Calomelanos gr. iij; pulv. jalapæ, gr. vj, 4tis horis.

9th. Took only part of two powders; several small heterogeneous stools; no evident alteration.

Habeat calomelanos gr. vj. 6tis horis.

10th. Two copious spinnage-looking stools, with a green inodorous mass resembling turtle fat, of about an ounce in weight; has taken the calomel regularly; holds up his head and looks about; will suffer me to feel his pulse, 110 to 120, cannot be certain. Tongue little furred, rather white; has only cut the incisores and one other tooth; the mouth has been hot and dry during the last week: cannot discover any tooth pressing forwards.

Continuentur calomelanos doses.

11th. More green stools; seems better; pulse 96 to 100.
Cont. calomel.

12th. Very sick in the night; vomited; only one stool, small and greenish; lays back his head as formerly; knits his brows; turns from the light. I fear hydrocephalus, and tell the mother so. I wish her to apply leeches to the nape, and a blister. She will not suffer the child to be tormented. Skin more hot than heretofore.

Continuentur calomel. et sumat. tinct. digitalis gtt. iv, post pulverem 6tis horis.

13th. Convulsed in the night; a little so now in the right arm; knits the brows; pupils contracted; mouth dry and hot; pulse 130, or rather more; skin rather hot; one cheek flushed; no unusual pulsation of the carotid or temporal arteries; the child lies quiet; no stool.

Persuaded the mother, by much intreaty, to apply six leeches to the nape. Pulv. jalapæ gr. vj, cum calomelane 6tis horis.

15th. The powders were not given, and the drops laid aside; the leeches did their duty; no stool.

I gave the child two powders at once, with much difficulty; it cried and screamed violently, and was convulsed after it lay down: one eye drawn.

16th. Had one large mottled stool yesterday afternoon, and made much water in the night; one pupil dilated; the right arm convulsed; no strabismus; pulse 130, unequal.

Gave him six drops of the tincture of digitalis, and requested the mother to repeat it every fourth hour.

17th. No stool; screamed violently this morning; both pupils dilated; strabismus left eye; pulse very small and frequent.

18th. Still alive; lying senseless; cannot swallow.

19th. Still alive; pulse not to be counted; strabismus both eyes.

20th. Died, much convulsed.

*Dissection, with much difficulty obtained.* On raising the calveria, found the appearance of the dura mater healthy; slit it down on each side of the falx, and divided the tentorium; disengaged the brain entirely, after the manner of Spurzheim. The cerebral veins and sinuses turgid; no inter-membraneous effusion. On dividing the medulla spinalis, as far down in its vertebral cell as the point of the scalpel would reach, some serous fluid escaped. Divided the several nerves at their points of egress from the cerebral substance, nearly in the order of their numerical arrangement. Turned out the brain entire upon a plate. The upper part of the medulla oblongata, the tuber annulare, and the crura cerebelli, covered with a gelatinous flaky matter. The third pair of nerves, and the optic nerves, thickly imbedded at their source in this substance. One of the optic nerves much altered in structure, harder and darker than the other. Slit open the ventricles, beginning at the fourth—found them all distended with colourless fluid; the plexus choroides in the fourth, or lateral ventricles, pale; sliced the brain, the substance entire, but rather soft round the ventricles. Abdominal examination not permitted.

Case II. On the 27th of November, 1816, I was called to Master Y——, who complained much of his forehead; impatient and irritable; the eyes expressive of aversion; corrugation between the eye-brows; no convulsive motions, excepting about the mouth; no pain of ancle or belly. Felt pain on making pressure between the tendons of the trapezius; but he was irritable, and no reliance could be placed on this. The apothecary had purged him well with calomel, and had applied three or four leeches to the temples. He never had vomiting.

Ordered twelve leeches to the nape; head to be shaven; vinegar and water applied on napkins constantly; blisters to the inside of the thighs, and a purge of calomel, gamboge, and jalap.

28th. Had many watery, and some spinnage-like stools, and passed one lumbricus in the night. Head easy; complains much of his bowels; eyes look natural; no twitchings about the mouth; pulse 98.

An almond mixture, with ten drops of the tincture of digitalis, every fourth hour.

29th. No stool; pain of belly continues; no pain of head; no convulsive motions any where; pulse 90. Rep. purg. of calomel, gamboge, and jalap, and continue the mixture.

30th. Several loose and greenish stools, but other symptoms as yesterday; pulse 84. To desist the purging, to see how far the appearance of the alvine evacuations depend on the purging ingredients. Continue the mixture.

December 1st. One stool of some consistence, and although not quite homogeneous, yet rather of healthy appearance. No pain any where, nor convulsive motions; eyes look natural; thought him in a fair way of recovery; pulse 80 to 84. Three hours afterwards, being 12 at noon, he became suddenly and generally convulsed, screaming violently at intervals. I did not see him until the evening; his eyes had precisely the appearance that struck me on the first day of my attendance. He now complains of his head; pulse 120.

A dozen leeches to the nape, and a continuance of the mixture, with digitalis.

2d. Amaurosis right eye; catching in breathing; pulse 140 to 160.

Consider the case lost, effusion having taken place; but to continue the digitalis, twelve drops every fourth hour.

3d. Violently convulsed, and delirious in the night; singing and screaming alternately; no stool; has not made water during twenty-four hours. Semicupium—fell asleep in it; made water. Digitalis.

4th. Has not been convulsed since in the bath; the pupil of the right eye continues dilated, and insensible to the rays of a candle; the other eye sensible of the light.

5th. Died: and in the evening examined the head.

*Dissection* Dura mater healthy—being slit close on each side of the falx through the tentorium, the medulla oblongata divided, and the base of the brain raised, find the tuber annulare and adjacent parts imbedded in a gelatinous substance, which surround the nerves at their origin, and with the optic at the front of decussation, forms a confused mass, approaching the appearance of genuine coagulated lymph. Much fluid in all the ventricles; the substance of the lobes sound.

Case III. March 4th, 1817. J. S. ætat. 8, about a week ago, received a kick in the left side; he felt sick immediately after it; in a day or two became feverish, and complained of his head. Visiting another person in the house, he was shown to me to day. Aversion to light; impatient of touch; head-ache, but not continued, sharp at intervals over the orbits. Neither pain nor discolouration where he received the kick; bowels rather constipated; tongue white; pulse 104. V. S. ad ℥viij.

℞. Hydr. submur. gr. iv; pulv. jalapæ gr. x. statim sumendus.

5th. Blood not buffed; bowels well evacuted; no headache; much languor; disinclination to food and exercise. No medicine; light and spare diet.

8th. Did not see him again until this day. No stool to-day or yesterday; listlessness; aversion to food; querulous; lays down his head; a dull appearance of the eye; no suffusion; no determined head-ache; tongue not foul; pulse 120, not beating uniformly. Do not like his appearance.

℞. Hydr. submur. gr. iv.; pulv. jalapæ gr. x. fiat pulvis 4tis horis sumendus. Mitte, No. vj.

9th. Took four of the powders, vomited the second; several dark mottled stools, inclining to green; much pain of bowels, and of the left arm; pupils contracted; slight aversion to light; face not flushed; no inordinate pulsation of the carotids, or temporal arteries. He will not acknowledge head-ache; is dull and quiet, if let alone; but if touched, rouses himself up, and speaks angrily and fretfully. Pulse 120 to 130, small and unequal; not much heat of skin; tongue moist, pretty clean; no thirst. Have not observed him to contract his brows, but the mother says he does.

Applicentur hirudines xij inter columnas colli, et continuentur pulveres 6tis vel 8vis horis, ut purgetur alvus.

10th. Several spinnage-like stools; leeches bled freely; more himself to-day; sits up in bed tying a cord; complains of his bowels; says he has a pain at the back of his head; pulse little altered; tongue pretty clean; eyes more expressive of natural feeling. Omit. pulveres.

Sumat tincturæ digitalis, gtt. xv. quartis horis.

11th. Lay quiet all night; heavy and quiet now; says that he has not any pain, but it is difficult to make him answer. One cheek a little flushed; eyes not suffused, but does not like to open them; knits his brows; pulse 120, small, not sharp, unequal; heat of skin trifling; back part of head evidently hottest.

Repeat the application of twelve leeches: afterwards shave the head, and apply linen cloths wetted with distilled vinegar, and water to the whole surface. Gave one of the cathartic powders, with directions to repeat it if necessary. Continuentur tinctura digitalis.

12th. Took two powders; had one large spinnage-like stool; lies quiet; complains by a sort of scream. On being closely questioned, says it is his bowels: looks pale; eyes unsteady; pupils appear natural; upper eye-lids drop; pulse 112 to 120, unaltered in character; very little febrile heat.

Cold application to the head and digitalis continued.

13th. Screamed violently in the night; with difficulty they kept him in bed; made no water; one green stool; eyes not suffused, but glassy; pupils contracted; right arm observed to twitch.

Emplastrum lyttæ ad cervicis spinam. Cold application to the head, and digitalis continued.

14th. Quiet, and seems considerably relieved; looks about with apparent attention; asked for some tea and bread, and took it; pulse 84 to 90, and more open; no stool. Says the pain is in his head.

Continue the cold application and digitalis.

15th. Attacked with a general convulsion this morning, which lasted half an hour; now laying quiet and stupid; both pupils dilated; pulse 140 or more, indistinct. He will not swallow any thing.

Warm bath. No medicine. Discontinue the cold application.

16th. Had a convulsion in the bath; since which, strabismus. Now laying in a stupor; pulse beyond 150; right arm moving in jerks; passed fæces and urine involuntarily.

17th. Died this morning, violently convulsed.

*Dissection.* Opened the head and body in the evening of the 17th. No fluid between the meninges; the cerebral veins turgid, and the sinuses very pale. Turned out the brain entire upon a plate; some fluid escaped in dividing the medulla oblongata. The pia mater highly vascular in its posterior part. The tuber annulare involved in a mass of coagulable lymph, or something approaching to it. A small collection of greenish purulent matter in the left lobe of the cerebellum; the origin of the nerves altered in colour and consistence, being darkened and hardened. All the ventricles nearly filled with serum; the plexus choroides pale; the lobes of the cerebrum and their great anterior commissure sound. The abdominal viscera healthy. No trace of injury from the kick.

---

I am sensible of the many points unattended to, in noting the symptoms in the cases here presented; and also in the anatomical research. In old times we were content with slicing down the brain, until we exposed the lateral ventricles, and saw the water; here the labour rested, supposing the cause ascertained: for the same reason, perhaps, I have taken little trouble beyond the examination of the state of the base of the brain.

I am, indeed, indebted to an able naval surgen settled in this city (Mr. Henderson), for making the dissections here detailed; and many others subsequent, of the same disease, whose history, in some instances, was not preserved; in others, not known to me, beyond one day or two previous to death: and if he has not been sufficiently minute in his research, it is because, at the time of making the dissection, we thought it irrelevant. But although much pains have been taken by Quin, Fothergill, Rush, Cheyne, and other celebrated men, to place the pathology of this disease on a firm basis, still the investigation of the subject is imperfect. Many years have elapsed since I read those authors whose names have been just mentioned, for in drawing up this paper I have purposely avoided the subject, as much as possible in my reading, that I might the better present my own observations and opinions, such as they are, towards the perfecting of our knowledge on this insidious and fatal disease. But it is to be hoped, that he who lately has given us reason to believe that "infantile remittent fever" is dependent on a peculiar sub-acute inflammation of the spinal cord, will trace it, and hydrocephalus acutus, by patient investigation, and close morbid anatomical research, through their several similitudes and discrepancies, showing their respective dependencies upon the diseased states of the spinal and cranial brains; and perhaps, at last, he may demonstrate that these two diseases, so much alike in character, are more alike in cause, than we had hitherto imagined.

---

But to return to my own paper. I have said, that the fatal link of the chain of morbid action, in the disease under investigation, is an inflammatory condition of the posterior arteries of the encephalon; and that this is the point to which our attention should be directed in the modus medendi.

We have seen in the dissections just detailed, an abundance of gelatinous matter and coagulable lymph covering the pons Varolii and adjacent parts: there can be no hesitation in pronouncing this appearance the product of inflammatory condition; and I must add, that in fourteen brains, whose subjects died of this disease within the last four years, I did not, in one instance, find this appearance wanting. The next most frequent phenomenon is the effusion into the ventricles; and this too is a necessary fruit of sub-acute inflammation of the basilar artery, when extended to its branches; the minute ramifications of which spread themselves upon the surfaces of the ventricles. But, that parts supplied by some of the posterior ramifications of the internal carotids should frequently be involved in this disease, must necessarily happen when the inflammatory condition becomes extended, seeing that the posterior cerebral artery anastomoses with the basilar: and that phrenitis also should occasionally be subjoined, in an inferior degree, must sometimes happen from contiguity of parts; but I believe this occurrence by no means frequent. And it has struck me, when standing over the sick bed, that the excited temporary flushing of one or both cheeks, or of the whole face, combined with the other appearances incident to patients labouring under acute hydrocephalus, have induced a belief in the identity of this disease and phrenitis. Look at the state of the myninx in our dissections; there is not a trace of phrenitic inflammation; observe the temporal arteries of your patient; they are quiet: not so in idiopathic phrenitis, or when it obtains the middle stage of tropical or of typhous fevers.

When effusion is found between the membranes in acute hydrocephalus, it is between the pia mater and tunica arachnoidea; the latter of which is raised up in different places by the fluid. When it takes place in fever, and in true phrenitis, it is found between the dura mater and the arachnoid membrane, and immediately in contact with the bony parietes of the cranium.

I think it unnecessary to pursue the argument in proof of the state of the encephalon and its vessels in hydrocephalus acutus; sufficient is made out to show its particular and distinct existence.

To insist upon its inflammatory nature is waste of time: on this point, I believe, all men are agreed on this side of the channel; but not precisely so, as to the circumstances under which the inflammatory condition obtains.

"In children, (says Spurzheim) the brain increases rapidly; hence there is naturally a great determination of blood to the head, and any febrile affection will increase that determination." But it has been said by many writers of reputation, to be dependent on a strumous diathesis for its existence The disease certainly is most frequent in families where that constitution may be recognized; and many children of the same family, in some instances, fall under it, each about the same age. Glandular swellings and tubercular phthisis manifest themselves frequently in individuals of the same family, at a particular period of life, differing as to the period in different families. Here we perceive analogy; but I cannot discover any thing like scrofulous condition in the diseased parts. I have seen scrofulous tubercles in the substance of the brain, and they suppurate as they do in the lungs; I believe the greenish collection of matter in the cerebellum, noticed in the third dissection, to have been one of them. I have seen them in the lobes of the cerebrum; and I recollect, in the case of an infant who died in consequence of a suppurated tubercle in the anterior lobe of the cerebrum, that the lungs were found studded with quiescent tubercles resembling grains of Paradise; the mother and her family became the victims of tubercular phthisis. But the state in which the base of the brain is found in all cases, truly pathognomonic, does not exhibit any thing like what we see in scrofulous inflammation any where else. Still I dare not conclude that it is not so, because I believe the essence of scrofula to consist in imperfect organization of structure, be it where it may; and that when inflammation obtains in such a part, the product is modified accordingly, and so it may be at the base of the brain. But inflammation is not less so in nature, although it may in degree, where the part under its influence has become imperfect in structure and excitability by any previous constitutional malady.

Whatever the nature of the inflammation may be, we must nevertheless keep in mind, during our whole treatment, that it is inflammation; and that the effusion is nothing more than one of the results of its presence, and that it has its seat in the posterior arteries at the base of the brain.

It has been remarked somewhere, that, in dissection of the brain after acute hydrocephalus, the vascularity is not so great as might be expected from the violence of the symptoms, were they dependent for their existence entirely on the inflammation. This observation loses all its weight, when we recollect that the vessels have been relieved by the effusion previous to death, exhibiting only traces of that distention which they previously had obtained.

To attempt any thing curative after effusion has taken place in hydrocephalus acutus, I consider idle. In phrenitic effusion, absorption may take place, and recovery supervene. Instances of such recovery have occurred under the eye of Dr. Dickson, of Clifton, when on service, as Physician to the Fleet; at least, he had strong reasons to draw such conclusion: and the medical world cannot but join me in attaching the greatest importance to the opinion of that gentleman, seeing that of such a fact we cannot have ocular demonstration, and knowing his ability as a physician and pathologist. But in hydrocephalus acutus, it is impossible that recovery should follow absorption of the effused serum, because the very fountain-head of sensorial power has laboured under previous vascular extension, to the destruction of its integrity. No such injury arises from inflammation of the myninx, and such other parts as receive their supply from the branches of the external carotid. Here is a practical distinction between the diseases that this essay would separate.

Doubts have arisen as to the fact of absorption ever taking place in hydrocephalus acutus. I incline to the sceptics: first, because anatomy cannot discover absorbents within the arachnoid envelope; and again, because the hydrops cerebri never disappears by the process of absorption; and again, because the substance of the brain does not suffer diminution in marasmus. And if we do not detect the event in dissection, it cannot obtain with recovery; as, previous to the effusion, there has been sufficient done to destroy life.

Since I determined upon making this communication, I have anxiously desired to examine the spine of a child who had died of hydrocephalus acutus; but although two fatal cases have recently presented themselves, permission could not be obtained. The same mistaken fondness which prevented the application of the wisest measures, refused the inspection of the body. The dissections of Dr. Sanders, given to us in the Medico-Chirurgical Journal for January last, have inspired this desire. His researches into the state of the spinal marrow and theca vertebralis in general fevers, are sufficient to induce a suspicion that this organ seldom escapes a diseased condition, in any general conflict of the system, whether with a contagious poison from without, or a disturbed equality and freedom of the circulation from within.

Leisure, and great opportunities for clinical observation, are necessarily opposed to each other; and perhaps it has been well observed, that there are but two periods in the life of a physician, at which he should write. On physiology, before he is engaged in full practice; on pathology and therapeutics, when he retires from the active and laborious duties of his profession.—In drawing up this paper, trifling as it is, I have felt the force of the observation; for one hour is the longest period I have been able to devote to it. There are men who go through the fatigue of considerable practice, and at the same time produce entire and admirable books, with astonishing ease; such men of the present day are Armstrong and Johnson: and the latter, like a vigorous pedestrian, who, walking fifty miles a day for twenty days, took a turn or two in the garden for amusement before and after his task, conducts a Journal that may not yield to any, as far as it is dependent on the Editor, for spirit and intelligence.

Nearly every thing I shall offer therapeutically, will have been anticipated by the tendency of my pathological views; I will not, however, cut the matter too short, but speak of such means as have appeared to me the most useful upon trial.

The torpid state of the bowels, and the morbid condition of their evacuations have attracted much attention; and by some writers, and by almost all practitioners, have been considered as the first link in the chain of the disease; but on dissection we find nothing; it is only disordered function: not so in the brain. We therefore may consider the state of the bowels to be dependent on the irritation which the par vagum is sustaining at its source in the medulla oblongata; or on some other indirect influence from the obvious seat of the disease.

From this view of the case, I have ceased in my practice to pursue the use of purgatives beyond their ordinary exhibition, so as to relieve the bowels daily, after having freely evacuated them at the onset; and as to the spinnage-like stools, I am convinced that they are the genuine product of the action of the calomel on the mucous follicules of the intestinal canal. The nausea and occasional vomiting are also sympathetic.

The point then claiming immediate attention, when the diagnostic symptoms are recognized, or even strongly suspected to be present, is the state of the encephalon. Leeches should forthwith be applied to the nape, in number from six to twenty-four, according to the age of the patient; and after they have done their office, a narrow blistering plaster should be applied over the four lower cervical and two upper dorsal vertebræ. By the timely application of these means, the patient shall sometimes evince an amendment, followed by recovery, that will force you to doubt the reality of your suspicions: but in this there is no great harm—too generally the amendment is ephemeral. The head is to be shaven, and oxycrate assiduously applied to its whole surface upon a linen napkin. Leeches must repeat their attack upon the intercolumnal fossa of the neck, until a fixed paleness of the child's lips forbids their employment. The blister should be kept open with savine cerate, and the bowels gently moved, avoiding all unnecessary irritation from much calomel and other drastic purgatives.*

This plan may be pursued until recovery dawn upon us, or until the coming of that delusive stage of the disease which simulates recovery, by the temporary relief afforded during effusion. If recovery be the result, the event soon becomes evident, and bark is the best medicine to secure the victory; but, should the stage of stupor supervene, we may shut up the Materia Medica.

I have given up the use of digitalis in this disease; I never could discover any beneficial effect from its exhibition. Calomel too, excepting as a purgative at the onset; and all mercurials I consider worse than useless. General bleeding I have not found to answer; it weakens the patient, without making a corresponding impression on the disease.

Every one has witnessed the slow but persevering progress of scrofulous inflammation in the glands of the neck, in the groin, and in the joints; and how reluctantly it yields to the application of leeches and other means found to be effectual in ordinary inflammation. Yet we are obliged to use those means, not being acquainted with any others so efficient: and the same apology may be offered for the methodus medendi here recommended to our perseverance, in the treatment of hydrocephalus acutus.

*Bristol, July* 20, 1818.

---

* In the midst of apparent health, children sometimes are attacked with sickness and convulsions, induced by much eating, by lumbrici, by retained fæces, or other intestinal irritants; after the fit, they lie in a comatose state, with dilated pupils, attended by strabismus. The patient has all the appearance of one in the last stage of hydrocephalus, but the pulse is generally slow; and these formidable appearances succeed a state of health so rapidly, that a moment's reflection will enable a medical practitioner, suddenly called in, to discriminate between the disease before him, and that which it simulates. And he will have the satisfaction to find, that the abstraction of a little blood will rouse his patient; and the subsequent administration of a cathartic shall reclaim him from the apparent danger in which he seemed to be placed.

