# Erratum

**Published:** 1819-01-01

**Authors:** 


### ERRATUM.

In Dr. Porter's paper on Hydrocephalus, page 284, last line, omit *inverse*.

